# Varenicline Targets the Reinforcing-Enhancing Effect of Nicotine on Its Associated Salient Cue During Nicotine Self-administration in the Rat

**DOI:** 10.3389/fnbeh.2019.00159

**Published:** 2019-07-17

**Authors:** Vernon Garcia-Rivas, Jean-François Fiancette, Nazzareno Cannella, Maria Carbo-Gas, Prisca Renault, Jessica Tostain, Véronique Deroche-Gamonet

**Affiliations:** ^1^Psychobiology of Drug Addiction, NeuroCentre Magendie, INSERM U1215, Bordeaux, France; ^2^University of Bordeaux, Bordeaux, France

**Keywords:** intravenous self-administration, nicotine, cues, individual differences, varenicline, rat

## Abstract

Nicotine is acknowledged as the key addictive compound of tobacco. Varenicline (Champix^®^ or Chantix^®^), mainly acting as a partial agonist at the α4β2 nicotinic receptor, is an approved smoking cessation pharmacotherapy, although with efficacy limited to a portion of smokers. Smokers differ in the motives that drive their drug seeking and Varenicline might be more efficient in some groups more than others. Studies in rodents revealed that nicotine-seeking is strongly supported by complex interactions between nicotine and environmental cues, and notably the ability of nicotine to enhance the reinforcing properties of salient environmental stimuli. It is not yet understood whether the decrease of nicotine-seeking by acute Varenicline in rats results from antagonism of the primary reinforcing effects of nicotine, of the reinforcement-enhancing effect of nicotine on cues, or of a combination of both. Thanks to a protocol that allows assessment of the reinforcement-enhancing effect of nicotine on cues during self-administration in rats, we showed that Varenicline targets both nicotine reinforcing effects and reinforcement-enhancing effect of nicotine on cues. Importantly, individual variations in the latter determined the amplitude of acute Varenicline-induced decrease in seeking. These results suggest that Varenicline might be more beneficial in smokers who are more sensitive to nicotine effects on surrounding stimuli.

## Introduction

Tobacco dependence continues to be a worldwide health burden, being responsible for as many as 7 million deaths per year (WHO, 2017). More than 70% of smokers wish to quit (U.S. Department of Health and Human Services, 2012), but less than 10% succeed without medical support (Rigotti, 2012). Even so, a major obstacle in ceasing to smoke is the limited efficacy of available treatments against tobacco dependence (Schuit et al., 2017). For instance, from all patients treated with Varenicline (Champix^®^ or Chantix^®^), one of the most effective approved pharmacotherapies in supporting smoking cessation (Cahill et al., 2013; Hartmann-Boyce et al., 2014), only 40% remain abstinent at the end of a 12-week-long treatment, while post-treatment abstinence rates drop to 20% in the following months after treatment cessation (Oncken et al., 2006; Niaura et al., 2008; Jordan and Xi, 2018).

Varenicline is a full agonist at the α7-, and a partial agonist at the α4β2-containing nicotinic cholinergic receptors (Coe et al., 2005; Rollema et al., 2007a,b), which mediate the primary reinforcement properties of nicotine, the major psychoactive compound of tobacco (Benowitz, 1992). However, the relatively weak primary reinforcement of nicotine cannot explain the pervasiveness of tobacco abuse alone (Caggiula et al., 2001; Rose, 2006). Recent studies have highlighted that nicotine can increase the reinforcing value of environmental cues that are primary reinforcers by themselves, or that have acquired reinforcing value through pairing with another reinforcer (Caggiula et al., 2009; Rupprecht et al., 2015). The interplay between nicotine and environmental cues is complex and difficult to disentangle, but plenty of evidence suggests it is a determinant factor in tobacco seeking (Caggiula et al., 2001, 2002; Garcia-Rivas and Deroche-Gamonet, 2019). Importantly, newer evidence suggests that smokers differ in the psychobiological mechanisms that drive their nicotine-seeking (for review, see Garcia-Rivas and Deroche-Gamonet, 2019). In this regard, understanding the psychopharmacological dimensions of nicotine-seeking that are being affected by Varenicline could clarify its limited efficacy. However, the numerous studies that have shown that Varenicline can acutely decrease nicotine self-administration in rodents (Rollema et al., 2007b; O’Connor et al., 2010; Le Foll et al., 2012; Funk et al., 2016), have done so in experimental conditions that do not clearly allow the disentangling of the psychopharmacology of Varenicline against nicotine and nicotine-cue interactions.

Furthermore, even though the effects of Varenicline on nicotine-cue interactions have also been subject to extensive studies (Levin et al., 2012; Schassburger et al., 2015; Barrett et al., 2018), they have been studied in conditions under which nicotine is not self-administered. Varenicline has been shown to dose-dependently antagonize the reinforcement-enhancing effect caused by nicotine (Levin et al., 2012). Consistent with its nature as a partial agonist, it has also been shown that Varenicline can enhance responding for a visual cue in a dose-dependent manner, although with a much weaker effect than nicotine (Barrett et al., 2018). This last result is consistent with a previous study, which used self-administration of Varenicline and a visual cue self-administered through two different levers, to reveal such reinforcement-enhancing effect of Varenicline (Schassburger et al., 2015). However, since the psychopharmacological actions of Varenicline in humans are of therapeutic relevance when nicotine intake is volitional, the testing of Varenicline effects on passive nicotine administration has weaker face validity when compared to the classical approach of drug self-administration (Panlilio and Goldberg, 2007).

Thus, the precise psychopharmacological mechanisms through which Varenicline opposes nicotine self-administration in rodents is still not well understood, but warrant further investigation. Because a key determinant of the synergistic interaction between nicotine and a salient cue is the primary reinforcing effects of the cue (Chaudhri et al., 2006; Caggiula et al., 2009), we developed an experimental procedure that allows for increasing these primary reinforcing effects during self-administration and tested the effect of Varenicline while contingently manipulating the reinforcing-enhancing effect of nicotine on the cue.

## Materials and Methods

### Subjects

Male Sprague–Dawley rats (Charles River, France), weighing 280–300 g at the beginning of the experiments, were single housed under a 12 h reverse dark/light cycle. In the animal house, temperature (22 ± 1°C) and humidity (60 ± 5%) were controlled. Rats were habituated to environmental conditions and experimental handling for 15 days before surgery. Standard chow food and water were provided *ad libitum*. All procedures involving animal experimentation and experimental protocols were approved by the Animal Care Committee of Bordeaux (CEEA50, N° 50120168-A) and were conducted in accordance with the guidelines of the European Union Directive 2010/63/EU regulating animal research.

### Surgeries

A silastic catheter (internal diameter = 0.28 mm; external diameter = 0.61 mm; dead volume = 12 μl) was implanted in the right jugular vein under ketamine (80 mg/kg)/xylazine (16 mg/kg) anesthesia. The proximal end reached the right atrium through the right jugular vein, whereas the back-mount passed under the skin and protruded from the mid-scapular region. Rats were given 5–7 days recovery before nicotine self-administration training began.

### Drugs

Ketamine hydrochloride (80 mg/kg; Imalgène 1000; Rhône Mérieux, Lyon, France) and xylazine hydrochloride (16 mg/kg; Rompun; Rhône Mérieux, Lyon, France) were mixed with saline and administered intraperitoneally in a volume of 2 ml/kg of body weight. (-)-Nicotine-Hydrogen-Tartrate (Glentham, UK) was dissolved in sterile 0.9% physiological saline for a final dose of 0.04 mg/kg free base. Nicotine, as well as sterile 0.9% physiological saline in control groups, was self-administered by the rats *via* intravenous (i.v.) route in a volume of 40 μl per self-infusion. Nicotine solution was adjusted to a pH of 7.

Varenicline or 7,8,9,10-Tetrahydro-6, 10-methano-6H-pyrazino[2,3-h] benzazepine tartrate (Tocris, UK) was dissolved in sterile 0.9% physiological saline for a final dose of 1 mg/kg free base. Varenicline was administered intraperitoneally (i.p.) 30 min prior to self-administration, in a volume of 2.5 ml/kg.

### Intravenous Self-administration

#### Self-administration Apparatus

The self-administration setup consisted of 48 self-administration chambers made of plexiglas and metal (Imetronic, France), and equipped with holes as operant manipulanda. Each chamber (40 cm long × 30 cm width × 36 cm high) was located in an opaque sound-attenuating cubicle equipped with an exhaust fan to assure air renewal and mask background noise (Supplementary Figure S1). For self-administration sessions, each rat was placed in one chamber where its chronically implanted intravenous catheter was connected to a pump-driven syringe (infusion speed: 20 μl/s). Two holes, located at opposite sides of the chamber at 5.5 cm from the grid floor, were used to record instrumental responding. In given experimental groups and experiments, a common white light (white LED, Seoul Semiconductor, South Korea), 1.8 cm in diameter, located 11.5 cm above the active hole, was used as nicotine (or saline) delivery-associated discrete visual cue, and is named thereafter “cue light” or “cue.” It produced 5 Lux. As well, in given experimental groups and experiments, a blue light (blue LED, Sloan Precision Optoelectronics, Switzerland), 1.8 cm in diameter, located on the opposite wall at 17 cm of the floor on the left side, was used as, and is named thereafter, “Ambient light” and abbreviated *AL*. It produced 15 Lux at a wavelength of 470 nm, which is known to not affect vision in Sprague–Dawley rats in a similar exposure pattern as in our experimental approach (Tosini et al., 2016). LED intensities were both measured in the middle of the cage with a Lux-meter (Moineau Instruments, France). Experimental contingencies were controlled and data were collected with a PC-windows-compatible SK_AA software (Imetronic, France).

#### Self-administration Procedures

**Figure 1:**
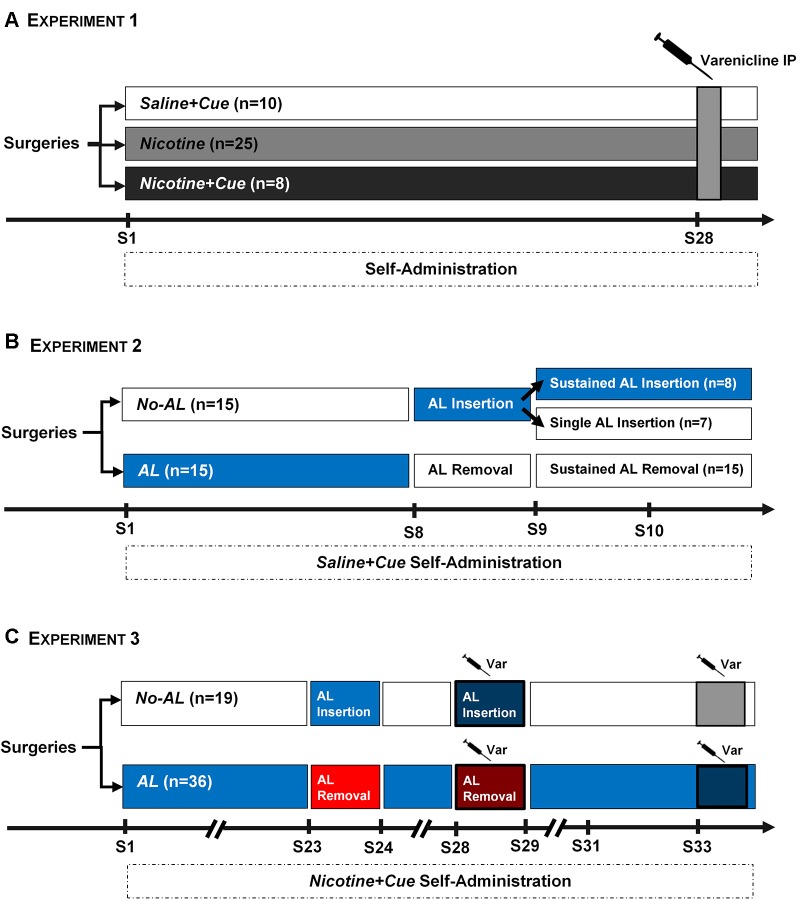
Experimental Protocols. **(A)** Experiment 1. Three groups of rats (*saline + cue*, *n* = 10; *nicotine*, *n* = 25; *nicotine + cue*, *n* = 8) were trained for self-administration for 27 sessions. The *Nicotine* group was substantially larger than the other two experimental groups, as it was expected that only around 40%–50% of animals in this group would acquire self-administration criteria. An acute IP injection of Varenicline was applied 30 min before session 28 of self-administration. **(B)** Experiment 2. Two groups of rats were trained for *saline + cue* self-administration. For one group (*AL*, *n* = 15), the Ambient light was on throughout the first seven sessions. For the other group (*No AL*, *n* = 15) the Ambient light was off during the same period. On the eighth session of self-administration, the Ambient light conditions were switched; removed for the *AL* group and inserted for the *No AL* group. On sessions 9 and 10, the *No AL* group was split into two, with half of the rats switched back to their original *No AL* condition (Single AL insertion, *n* = 7), while the other half remaining under the new *AL* condition (*Sustained AL insertion*, *n* = 8). All rats from the *AL* group remained without the Ambient light for sessions 9 and 10 (*Sustained AL removal*). **(C)** Experiment 3. Two groups of rats were trained for *nicotine + cue* self-administration, using the same *AL* and *No AL* conditions as in Experiment 2. The *AL* group was substantially larger (*n* = 36) than the control *No AL* condition (*n* = 19), as it was expected that *AL* could delay acquisition of *nicotine + cue* self-administration. Similar to Experiment 2, the *AL* conditions were switched in Session 23, after which rats were returned to basal conditions. On session 28, the switch of *AL* conditions was re-applied, with the addition of a Varenicline IP injection 30 min before session. Rats were then allowed to return to a stable baseline before a final test using a single Varenicline injection on a basal self-administration.

In the three experiments presented below, self-administration testing began 2 h after the onset of the dark phase. Nose-poke in the active hole under a fixed ratio three schedule of reinforcement (FR3) produced the activation of the infusion pump (40 μl over 2 s). Nose-pokes at the inactive hole were recorded but had no scheduled consequences. Rats in all protocols of self-administration described in this study were placed under an FR3 schedule of reinforcement from the first session onwards, with the reinforcer varying according to the experimental group in which they were allocated (Figure 1). Neither food-training nor FR-1 transition period was used. Whatever the reinforcer, rats were trained 2 h daily, 5 days per week, from Monday to Friday, except for the first session of Experiments 1 and 3 that took place on a Tuesday. To maintain catheter patency, catheters were flushed with ~10 μl of heparinized saline (30 IU/ml) after each self-administration session and before the self-administration sessions run on Monday.

In Experiment 1, to define a significant self-administration behavior at the individual level, we used a discrimination index between active and inactive holes [(active nose-pokes/total nose-pokes)*100] strictly superior to 50%, together with a minimal number of at least six self-infusions per session over three consecutive sessions and with stability in the number of self-infusions (±10%) over the last two sessions.

### Experimental Procedures

#### Effect of Varenicline on Self-administration Behavior Reinforced by Either a Discrete Cue Light, a Nicotine Infusion or a Combination of Both Nicotine and Cue Light (Experiment 1)

Nose-poking in the active hole at FR3 was reinforced either by an infusion of 0.04 mg/kg nicotine free base (*nicotine*, *n* = 25), a nicotine 0.04mg/kg infusion plus a discrete cue light (*nicotine + cue*, *n* = 8), or a saline infusion plus a discrete cue light (*saline + cue*, *n* = 10; Figure 1A). For the *nicotine* group, following nose-poking in the active hole at FR3 the infusion pump was activated for 2 s. For the *nicotine + cue* and *saline + cue* groups, nose-poking in the active hole at FR3 turned on the cue light located above the hole, simultaneous to the activation of the infusion pump. The cue light remained on for 4 s in total. Since it is known that nicotine alone is poorly self-administered in the absence of other salient stimuli (Caggiula et al., 2002), the *nicotine* group was substantially larger than the other two experimental groups, as it was expected based on preliminary data that only 40%–50% of animals in this group would meet the desired self-administration criteria.

After 27 daily basal sessions (Figure 1A), rats showing significant self-administration behavior were administered with Varenicline (1 mg/kg, ip) 30 min prior to a basal self-administration session. The average number of infusions over training sessions 26–27 was used as the baseline. The Varenicline dose was chosen based on previous literature (e.g., O’Connor et al., 2010).

#### Effect of Varenicline on the Reinforcement-Enhancing Effect of Nicotine During *Nicotine + Cue* Self-administration

##### A Procedure to Alter the Primary Reinforcing Effects of the Cue Light (Experiment 2)

A key determinant of the interaction between nicotine infusion and an associated discrete cue light relies on the primary reinforcing effect of the cue. A key issue is then to be able to manipulate the reinforcing effect of the cue during nicotine self-administration. The goal of Experiment 2 was to establish a protocol where the reinforcing effects of the cue can be altered. Therefore, we tested in rats self-administering *saline + cue* whether we could decrease or increase the primary reinforcing effects of the cue by altering its visual salience, by either adding or removing an interfering ambient light, respectively.

Two groups of rats were trained for *saline + cue* self-administration, as described in Experiment 1 except that for one group (*AL*, *n* = 15), the *Ambient light* (*AL*) was on throughout the first seven acquisition sessions. For the other group (*No Ambient light*, *No AL*, *n* = 15) the *AL* was off during the same period (Figure 1B). On the eighth session of self-administration, the *Ambient light* conditions were switched; turned off for the *AL* group and on for the *No AL* one. On sessions 9 and 10, the *No AL* group was split into two, with half of the rats switched back to their original *No AL* condition (*Single AL Insertion* subgroup, *n* = 7), while the other half remaining under the new *AL* condition (*Sustained AL Insertion* subgroup, *n* = 8). All rats from the *AL* group remained without the *AL* for sessions 9 and 10.

##### Effect of Varenicline on the Reinforcement-Enhancing Effect of Nicotine During Nicotine + Cue Self-administration (Experiment 3)

Based on the results of Experiment 2, two groups of rats were trained for *nicotine + cue* self-administration, as described in Experiment 1. As in Experiment 2 the *AL* was on throughout the basal training self-administration sessions, for one group (*AL*, *n* = 36), and was off for the other one (*No AL*, *n* = 19; Figure 1C). The *AL* group was substantially larger than the control *No AL* condition, as it was expected that the *AL* could delay acquisition of *nicotine + cue* self-administration. On session 23, we tested the effect of: (1) suppressing; and (2) adding, the *AL* on self-administration in the *AL* and *No AL* groups, respectively. Rats were then brought back to the respective basal conditions until session 28, when we tested the effect of Varenicline (1 mg/kg, i.p.) administered 30 min prior to session during which the *AL* was manipulated, i.e., suppressed in the *AL* group and inserted in the *No AL* group. Rats were then returned to basal conditions, and once responding was stable over two consecutive sessions and had returned to the level of infusions of sessions 21–22, we tested the effect of Varenicline (1 mg/kg, i.p.) administered 30 min prior to a basal session.

### Data Analyses

#### Self-administration

Total responses in the active and inactive holes and total number of infusions per self-administration session were considered.

#### Effect of Varenicline and/or *AL* Manipulation

To evaluate Varenicline and/or *AL* manipulation (*AL* removal or *AL* insertion), delta infusions from baseline (infusions at test − infusions at baseline) were calculated. Baseline infusions correspond to the mean infusions over the two sessions preceding a test.

### Statistical Analyses

Self-administration behavior was analyzed using repeated measures ANOVA with Time (number of sessions), Hole (active vs. inactive), Test (Baseline vs. Test), Condition (*AL*On to *AL*Off, *AL*Off to *AL*On, *AL*On to *AL*Off+Var, *AL*Off to *AL*On+Var), as within-subject factor, and experimental group (*saline + cue*/*nicotine + cue*/*nicotine*, *AL*/*No AL*) as between-subject factor.

Significant main effects or interactions were explored by pairwise comparisons of means using the Newman Keuls *post hoc* test. Pearson’s correlation analyses were used to investigate the correlation between variables of interest. A *t*-test was used to compare the *AL* Removal effects (or of *AL* Insertion effects) on *saline + cue* and *nicotine + cue* self-administration.

The results are presented as mean ± SEM. Differences were considered significant at *p* < 0.05.

The statistical analyses were performed using the STATISTICA 13.3.0 (2017) data analysis software system (TIBCO Software Inc., Palo Alto, CA, USA).

## Results

### Nicotine and a Cue Light Contribute Synergistically to Self-administration (Experiment 1)

**Figure 2:**
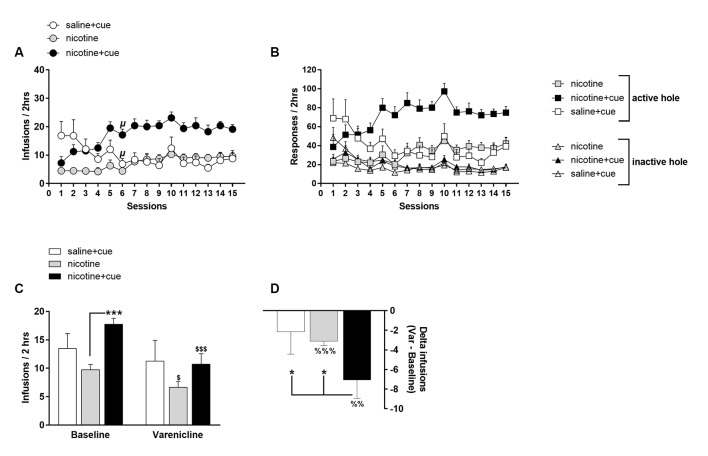
Nicotine and infusion-associated discrete cue light contribute synergistically to self-administration behavior. Operant nose-poking at FR3 in active hole was reinforced by the delivery of an intravenous infusion of saline associated with the lighting of a salient visual cue above the active hole (*saline + cue*), of a nicotine intravenous infusion associated with the lighting of a salient visual cue above the active hole (*nicotine + cue*) or of the sole delivery of a nicotine intravenous infusion (*nicotine*). **(A)** Infusions earned per session over the 15 first behavioral sessions. **(B)** Responses in the active and inactive holes per session over the 15 first behavioral sessions. Symbols denote group mean and error bars denote SEM. **(C)** Mean infusions earned in basal conditions (*Baseline*) and after Varenicline administration (1 mg/kg i.p., 30 min prior to session) in rats self-administering *saline + cue*, *nicotine + cue* or *nicotine*. For *Baseline*, infusions are averaged over the two last sessions prior to Varenicline test. **(D)** Effect of Varenicline as calculated by the delta between infusions earned in baseline and infusions earned under Varenicline effect, in rats self-administering *saline + cue*, *nicotine + cue* or *nicotine*. Symbols and bars denote group mean and error bars denote SEM. ^\rmu^*p* < 0.0001 as compared to respective session 1. *p < 0.05, ***p < 0.001. ^$^*p* < 0.05 and ^$$$^*p* < 0.001 as compared to respective baseline. ^%%^*p* < 0.01, ^%%%^*p* < 0.001, as compared to zero.

Over the first 15 self-administration sessions, *saline + cue, nicotine + cue* and *nicotine* rats differed significantly regarding number (Group, *F*_(2,42)_ = 10.77, *p* < 0.001) and pattern (Group × Session, *F*_(28,588)_ = 6.7, *p* < 0.0001) of reinforcers earned (Figure 2A), as well as number and discrimination in responses (Figure 2B).

Nicotine first tended to compromise, but secondarily amplified, the reinforcing effects of a discrete cue light. Thus, *nicotine + cue* rats increased self-infusions from session 1 to session 6 (*p* < 0.0001) while the *saline + cue* rats showed the opposite profile (*p* < 0.0001) when the *nicotine* rats remained stable over the same sessions (*p* = 0.87). The compared self-administration patterns of the three groups suggest that nicotine and cue interact synergistically.

### Nicotine and Saline + Cue Are Mild but Different Reinforcers (Experiment 1)

The behavior of the *saline + cue* and the *nicotine* groups stabilize at a similar level from session 6 (Figure 2A). Observations exclude, however, that the behavior is just driven by the stimulus that is common to the two groups, i.e., intravenous infusion. Indeed, up to session 6, the *saline + cue* group produced a higher number of self-infusions than the *nicotine* one (Group, *F*_(1,36)_ = 8.5, *p* < 0.01) and the two profile of self-infusions differ with decrease, and progressive increase, up to stabilization, respectively (Group × Session, *F*_(5,180)_ = 5.7, *p* < 0.0005). Also, in a preliminary experiment, eight rats were trained for *saline + cue* for 13 sessions in conditions similar to the ones described in Experiment 1. Omission of the cue on session 14 produced a significant decrease in self-administration (Supplementary Figure S2) supporting that the cue contributes to the reinforcing effects in *saline + cue* rats.

The mild reinforcing effects in *nicotine* and *saline + cue* rats, as compared to *nicotine + cue* rats, were further confirmed when using threshold criteria for discrimination, i.e., number of infusion and stability in behavior (see “Materials and Methods” section), to define a significant self-administration behavior at the individual level. By session 15, only 40% of the *nicotine* rats (10/25) had acquired self-administration, compared to 100% of the *nicotine + cue* rats (8/8), and 50% of the *saline + cue* rats (5/10; Supplementary Figure S3A).

Distribution of the individual scores of self-infusions in the rats showing self-administration based on these criteria (Supplementary Figure S3B) also further supports the difference in nature of the reinforcers acting in the *nicotine* and the *saline + cue* groups. Supplementary Figures S3C–F show the self-infusions and hole responses in rats, which either reached (Supplementary Figures S3C,D) or did not reach (Supplementary Figures S3E,F) these criteria.

### Varenicline Decreases *Nicotine + Cue* and Nicotine Self-administration (Experiment 1)

After 27 sessions, the effect of Varenicline on self-administration was tested in the *saline + cue* (*n* = 5), *nicotine + cue* (*n* = 8) and *nicotine* (*n* = 11) rats that met self-administration criteria evaluated on behavior during sessions 26 and 27. Varenicline decreased self-administration as measured by the number of self-infusions earned (Test effect, *F*_(1,24)_ = 30.6, *p* < 0.0001). This effect was function of the experimental group (Test × Group, *F*_(2,24)_ = 4.71, *p* < 0.05) with a significant effect in rats self-administering *nicotine + cue* (*p* < 0.0001) and *nicotine* (*p* < 0.05; Figure 2C). According to the effect on self-infusions, Varenicline decreased nose-poking in a group-dependent (Test effect, *F*_(1,24)_ = 22.49, *p* < 0.0001; Test × Group, *F*_(2,24)_ = 4.55, *p* < 0.05) and hole-dependent manner (Test × Hole, *F*_(1,24)_ = 28.4, *p* < 0.0001), exclusively targeting the active hole (Supplementary Figure S4).

The effect of Varenicline, as measured by the delta-infusions from baseline (Group effect, *F*_(2,24)_ = 3.29, *p* < 0.05), was higher in the *nicotine + cue* group than in the *saline + cue* (*p* < 0.05) and *nicotine* groups (*p* < 0.05), in which the delta-infusions were similar (Figure 2D). However, the effect of Varenicline was different from zero in the *nicotine* group (*p* < 0.0001), but not in the *saline + cue* group. Notably, in the nicotine group, the Varenicline effect, as measured by delta-infusions from baseline, did not correlate with basal self-infusions (**data not shown**).

### Varenicline Targets the Reinforcing-Enhancing Effect of Nicotine on Its Associated Salient Cue

Results of Experiment 1 supported that nicotine and the cue interact to produce reinforcing effects, and that Varenicline significantly decreased the *nicotine + cue* combined reinforcer. However, it did not allow concluding whether Varenicline was specifically targeting this interaction. To further explore this hypothesis, we aimed at testing the effect of Varenicline while manipulating this nicotine-cue interaction in the same individuals. As a first step, we aimed at developing a procedure that would allow promoting (vs. compromise) the nicotine-induced enhancement of the reinforcing properties of its associated cue. As this enhancement is depending on the primary reinforcing effects of the cue, we initially worked on a procedure allowing to increase (vs. decrease) these reinforcing effects.

#### An Interfering Ambient Light (AL) Appears to Alter the Primary Reinforcing Effects of the Discrete Cue Light (Experiment 2)

As in Experiment 1 rats self-administered *saline + cue*, as shown by a significant discrimination between active and inactive holes over the seven sessions of self-administration (Hole effect, *F*_(1,28)_ = 28.7, *p* < 0.0001; Supplementary Figures S5A,B). However, this discrimination was a function of the experimental group. The *AL* appears to compromise the expression of the reinforcing effects of the discrete cue light (Group effect, *F*_(1,28)_ = 10.4, *p* < 0.01). In standard conditions (*No AL*), *saline + cue* induced self-administration behavior, while in the *AL* condition, with the same *saline + cue* reinforcer, rats did not discriminate significantly between active and inactive holes (Group × Hole, *F*_(1,28)_ = 18.7, *p* < 0.0001). In the standard *No AL* condition, although behavior decreased over sessions, discrimination remained significant, up to the last session (*p* < 0.005).

**Figure 3:**
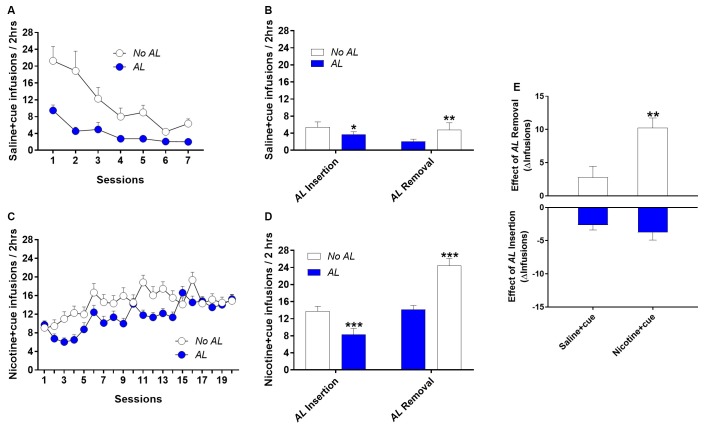
An interfering ambient light (AL) appears to alter the primary reinforcing effects of a salient discrete cue light. **(A)** Infusions earned per session over seven behavioral sessions during which operant nose-poking in the active hole was reinforced at FR3 by the delivery of an intravenous infusion of saline associated with the lighting of a cue light above the active hole. The presence of a 15 Lux *Ambient light* (*AL*) reduced self-administration behavior as compared to the control condition (*No AL*). **(B)** Effect on infusions earned of *AL Removal* and *AL Insertion* in rats trained for *saline + cue* self-administration over seven sessions in the *AL* and *No AL* conditions, respectively. Basal infusions are averaged over the two last sessions prior to *AL Insertion (or Removal)* test. The interfering AL delays acquisition of nicotine + cue self-administration. **(C)** Infusions earned per session over the first 19 behavioral sessions during which operant nose-poking in the active hole was reinforced at FR3 by the delivery of an intravenous infusion of nicotine associated with the lighting of a cue light above the active hole. **(D)** Effect on infusions earned of *AL Removal* and *AL Insertion* in rats trained for *nicotine + cue* self-administration in the *AL* and *No AL* conditions, respectively. The interfering AL procedure allows revealing the reinforcement-enhancing effect of nicotine on its associated salient cue. **(E)** Comparison of *AL Removal* and *AL Insertion* effects in rats trained for *saline + cue* or *nicotine + cue* self-administration. While *AL Insertion* in *No AL* rats produced a similar decrease in *saline + cue* and *nicotine + cue* rats (bottom), *AL Removal* produced a stronger increase in *nicotine + cue* rats (top). Symbols and bars denote group mean and error bars denote SEM. *p < 0.05, **p < 0.01, ***p < 0.001.

Not only *No AL* rats discriminated between the inactive control hole and the active hole associated with *saline + cue* delivery (Supplementary Figures S5A,B), but they also earned significantly more reinforcers than the *AL* rats (Group effect, *F*_(2,44)_ = 8, *p* < 0.01; Figure 3A).

It is unlikely that the absence of discrimination and the reduced number of reinforcers in *AL* rats was due to a non-specific stress-like or aversive effect. First, the number of inactive nose-poking was not affected (Supplementary Figure S5B), suggesting that the *AL* effect may be targeting the reinforcement of the cue light. Second, the switch of the *AL* conditions on session 8 further attested that the *AL* compromises the cue light reinforcing effects. While *AL Insertion* decreased self-administration, *AL removal* increased it (Condition × Group, *F*_(1,28)_ = 7.7, *p* < 0.01; Figure 3B).

To better understand the effect of *AL* Removal and Insertion, *No AL* rats were split into two groups for the following two sessions (9 and 10): one group (*Sustained AL Insertion*, *n* = 8), maintained the newly acquired *AL* condition, while the other (*Single AL Insertion*, *n* = 7) returned to their *No AL* condition (Figure 1B). *Sustained AL Insertion* further diminished self-administration in sessions 9 and 10, compared to sessions 6 and 7, while rats in the *Single AL Insertion* group appeared to compensate by increasing their mean infusions, when back to the initial *No AL* condition (Supplementary Figure S6). In the case of the *Sustained AL Removal* rats, the removal of the *AL* was maintained for sessions 9 and 10, further increasing self-administration in comparison to sessions 6 and 7 (Supplementary Figure S6).

#### The Interfering AL Procedure Appears to Reveal the Reinforcement-Enhancing Effect of Nicotine on Its Associated Salient Cue During Nicotine Self-administration (Experiment 3)

Having revealed that it was possible to increase the reinforcing effects of the cue by *AL* Removal, we tested its effect on *nicotine + cue* self-administration, both on acquisition and once behavior was established.

During acquisition under the *No AL* condition, the number of *nicotine + cue* self-infusions was higher than under the *AL* condition (Group effect, *F*_(1,49)_ = 5.36, *p* < 0.05), but the difference decreased over the 20 self-administration sessions (Group × Session, *F*_(19,331)_ = 4.14, *p* < 0.0001) and the *AL* group reached and maintained the level of self-infusions of the *No AL* group by session 15 (Figure 3C).

Rats in the *AL* condition did not discriminate between active and inactive holes in the first session, contrary to *No AL* condition (Supplementary Figure S5C). Even though inactive nose-poking was similar in the *AL* and *No AL* conditions from session 2, in a manner similar to *saline + cue* self-administration, active responding in the *AL* condition remained low compared to *No AL* conditions up to session 5.

Once stabilized, removal of the *AL* increased self-administration behavior by the *AL* group (Test effect, *F*_(1,35)_ = 47.9, *p* < 0.0001), while insertion of the *AL* decreased self-administration behavior by the *No AL* group (Test effect, *F*_(1,18)_ = 24.46, *p* < 0.001; Figure 3D).

As for *saline + cue*, it is unlikely that *AL* compromised *nicotine + cue* self-administration due to a non-specific stress-like or aversive effect. Notably, during the first self-administration session (Supplementary Figure S5C), total responses were not lower in *AL* rats, and absence of discrimination between active and inactive holes resulted from equal high responses in inactive and active holes, and not reduced responses in the active hole.

Critically, as summarized in Figure 3E, the effect of the *AL* removal was much more pronounced in *nicotine + cue* conditions compared to *saline + cue* conditions (*t*-test, *p* < 0.01), suggesting that any increase in visual salience of the cue is magnified by nicotine. By comparison, introduction of the *AL* had the same effect in both *nicotine + cue* and *saline + cue* conditions, suggesting a non-specific effect on visual perception, which is not potentiated by nicotine.

#### Varenicline Targets the Reinforcement-Enhancing Effect of Nicotine on Its Associated Salient Cue (Experiment 3)

**Figure 4:**
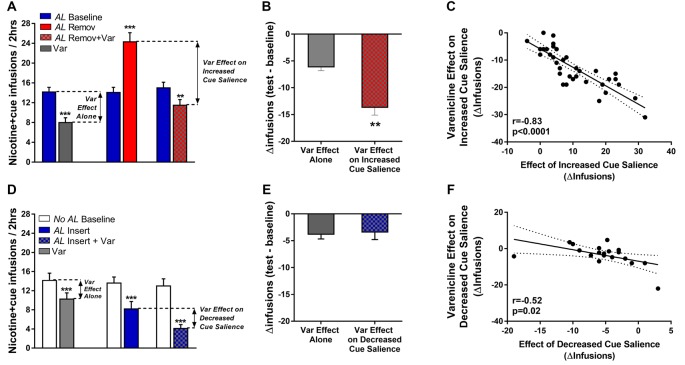
Varenicline targets the reinforcement-enhancing effect of nicotine on its associated salient cue. **(A)** Infusions earned in rats trained for *nicotine + cue* self-administration in the presence of the interfering *AL* (*AL Baseline*), in response to Varenicline (*Var*), to *AL* Removal (*AL Remov*) or a combination of both (*AL Remov + Var*). **(B)** Comparison of Varenicline effect in *AL Baseline* condition (Infusions *Var* *AL Baseline* − Infusions *AL Baseline*) and in *Increased Cue Salience* condition [by *AL Removal*; calculated from the combined effect of *AL Removal* and *Varenicline* (Infusions *AL Remov + Var* − Infusions *AL Baseline*) minus the effect of *AL Removal* (Infusions *AL Remov* − Infusions *AL Baseline*)]. Varenicline absolute effect was amplified in the *Increased Cue Salience* condition (by *AL Removal)*. **(C)** Almost 1 to 1 negative correlation between the effect of *Increased Cue Salience* and the calculated effect of Varenicline on *Increased Cue Salience*. The individual increase in *nicotine + cue* infusions by *Increased Cue Salience* was antagonized by Varenicline. **(D)** Infusions earned in rats trained for *nicotine + cue* self-administration in the absence of the interfering *AL* (*No AL* Baseline), in response to Varenicline (*Var*), to *AL* Insertion (*AL Insert*) or a combination of both (*AL Insert + Var*). **(E)** Comparison of Varenicline effect in *No AL* baseline condition (Infusions *Var* *No AL* baseline—Infusions *No AL Baseline*) and in *Decreased Cue Salience* condition [by *AL Insertion*; calculated from the combined effect of *AL Insertion* and *Varenicline* (Infusions *AL Insert + Var* − Infusions *No* *AL Baseline*) minus the effect of *AL Insertion* (Infusions *AL Insert* − Infusions *No* *AL Baseline*)]. Varenicline absolute effect was similar in the two conditions. **(F)** Correlation between the effect of *Decreased Cue Salience* [by *AL Insertion*] and the calculated effect of Varenicline on Decreased Cue Salience (by *AL Insertion*). Bars denote group mean and error bars denote SEM. Data points reflect individual scores. **p < 0.01, ***p < 0.001.

Once stabilized, self-administration behavior by the *AL* group was altered by removal of the *AL*, by Varenicline or a combination of both (Test effect, *F*_(2,70)_ = 64.8, *p* < 0.0001). According to the condition tested, the test effect was different however (Test × Condition, *F*_(2,70)_ = 76.3, *p* < 0.0001). *AL* removal alone produced an increase (Figure 4A, red bar) in *nicotine + cue* self-administration (*p* < 0.001). When *AL* removal was combined with Varenicline administration, Varenicline abolished completely the effect of *AL Removal* and decreased *nicotine + cue* self-administration below *AL* Baseline (Figure 4A, dashed red bar, *p* < 0.01 vs. *AL Baseline*). However, this latter effect was of a lower extent than when Varenicline was applied in the basal self-administration conditions, i.e., with maintenance of the *AL* (*p* < 0.001; Figure 4A, gray bar). Critically, Varenicline and *AL* Removal effects were not simply additive. When evaluating the effect of *AL Remov + Var* to the effect of *AL Remov* alone, one yields an effect which is much higher than the one of Varenicline alone on basal self-administration, suggesting that Varenicline specifically abolishes the enhancing effects of the *AL* Removal (Figure 4B). Noteworthy, this interpretation is supported by the correlation analysis (Figure 4C) showing a strong inverse correlation between the effect of *Increased Cue Salience* by *AL* Removal (Δ*AL*Remov = *AL*Remov − *AL* baseline) and the calculated Var effect during *Increased Cue Salience* by *AL* Removal (Δ*AL*Remov + var − Δ*AL*Remov). Varenicline treatment during Increased Cue Salience by *AL* Removal appears to reduce infusions from an amount equivalent to the increase produced by the Increased Cue Salience. In other words, in these *AL* Removal conditions, Varenicline appears to decrease specifically the individual increase produced by *AL* Removal, i.e., the individual potentiation of *nicotine + cue* self-administration produced by the Increased Cue Salience.

Self-administration behavior by the *No AL* group was decreased by insertion of the *AL*, by Varenicline or a combination of both (Test effect, *F*_(2,36)_ = 4.4, *p* < 0.05; Figure 4D). According to the condition tested, the test effect was different however (Test × Condition, *F*_(2,36)_ = 9.3, *p* < 0.001). Insertion of the *AL*, in rats trained in absence of it, produces a significant decrease in *nicotine + cue* self-administration (Figure 4D, blue bar), which was similar in amplitude to the effect of Varenicline (Figure 4D, gray bar). When combined with *AL* Insertion, Varenicline amplified the effect of the *AL* Insertion (Figure 4D, dashed gray bar). Notably, the combined effect of *AL* Insertion and Varenicline were not synergistic but additive as shown in Figure 4E. When subtracting the *AL Insert* effect from the *AL insert + Var* effect, to get the *Var effect on decreased cue salience*, the result was similar to the effect of Varenicline alone (*Var effect alone*; Figure 4E). Although less strong, similarly to the effect of Varenicline on *Increased Cue Salience* by *AL* Removal, there was a correlation between the decreased effect of *AL* Insertion on self-administration and the effect of Varenicline on this *AL* Insertion effect (Figure 4F), supporting that Varenicline had a bi-directional effect on the nicotine-induced increase cue reinforcement, depending on how the AL manipulation altered said cue reinforcement.

## Discussion

Varenicline is acknowledged as one of the most efficient therapeutic tools for tobacco dependence. However, its efficacy is limited both in time and to a portion of patients (Oncken et al., 2006; Niaura et al., 2008; Jordan and Xi, 2018). Even though the molecular pharmacology of Varenicline is well-known (Coe et al., 2005; Rollema et al., 2007a), its psychopharmacological actions are still poorly understood. In this study, we evidenced that acute Varenicline reduced nicotine-induced enhancement of the reinforcing properties of a nicotine-paired cue during intravenous self-administration. This effect appeared to depend on how much nicotine-cue interactions were contributing to self-administration behavior at the individual level. Conversely, the decrease by acute Varenicline of self-administration of nicotine alone appeared not related to individual basal levels of self-administration.

### Nicotine Alone Is a Poor Primary Reinforcer, but Is Strong Enough to Drive Self-administration in Certain Individuals, but Not in Others

Nicotine has weak primary reinforcement properties. Hence, classical nicotine self-administration has been developed to pair contingent nicotine IV delivery with the presentation of a salient visual cue light (Caggiula et al., 2002). A discrete cue light alone can act as a primary reinforcer in drug naïve rats (Deroche-Gamonet et al., 2002). In our study, we used the *saline + cue* condition as a control group evidencing the contribution of the cue in driving self-administration behavior. Comparison with the *nicotine + cue* group reveals the actual contribution of nicotine in *nicotine + cue* self-administration behavior.

In our study, by session 15, 100% of all rats trained in *nicotine + cue* condition showed criteria of significant self-administration behavior, but only 40% of all rats trained in the *nicotine* condition reached the same criteria. These results not only confirm the well-known observation described by Caggiula and colleagues, but it extends it with the observation that some rats appear much more sensitive to the reinforcing properties of nicotine, thus driving nicotine self-administration despite the lack of salient environmental cues, supporting that individuals may vary in the mechanisms that drive their nicotine-seeking (Garcia-Rivas and Deroche-Gamonet, 2019).

### A Novel Procedure That Allows Targeting the Reinforcing-Enhancing Effects of Nicotine on Its Associated Salient Cue During Nicotine Self-administration

In a previous study, Palmatier et al. (2007) demonstrated that the reinforcement-enhancing effects of nicotine on visual cues are dependent on the strength of the primary reinforcement of such cues in a nicotine-naïve state, with a stronger enhancing effect observed for visual cues with higher primary reinforcement properties. Further studies have assessed the effect of Varenicline on this nicotinic enhancement of cue reinforcement, but in conditions that are different from volitional nicotine intake (Levin et al., 2012; Barrett et al., 2018). Here, we developed a novel experimental approach that attempted a sudden increase in the visual salience of the nicotine-paired cue, through the removal of an interfering Ambient light (*AL*). This approach allowed us to explore the observations by Palmatier et al. (2007), but in the context of nicotine self-administration, and within the same individuals.

A possible explanation for the interfering effect of the *Ambient Light* *(AL)* in seeking behavior could be a non-specific aversive or stressful effect, rather than a reduction in the reinforcing effects of the cue. However, this explanation appears unlikely. The aversive effect of an ambient stressor would have impacted both active and inactive responding, while this is not the case. Critically, in the first *nicotine + cue* self-administration session, total responding was similar whether the *Ambient Light* was present or not. It is noteworthy that the presence of the *AL* delayed the acquisition of self-administration of *nicotine + cue*, which became equivalent to that of the *No* *AL* condition starting session 17. Overall, this data suggests that the effect of the *AL* is due to a reduction of the visual salience of the cue through visual interference, rather than a mere stress effect caused by the *AL*. Further studies, including progressive ratio schedules of reinforcement, could validate the interfering role of *AL* in cue reinforcement.

Importantly, the increase in self-administration due to removal of the visual interference was much more pronounced in *nicotine + cue* conditions compared to *saline + cue* conditions, supporting a nicotine-specific effect. This difference could be explained by the different value of the cue in these two conditions. In the *saline + cue* condition, the cue is acting as a primary reinforcer (Deroche-Gamonet et al., 2002). In the *nicotine + cue* condition, the cue is both a primary and a secondary reinforcer, and both reinforcing effects can be enhanced further by nicotine itself (Caggiula et al., 2009). However, it is more likely that the strong nicotine-specific increase in responding after *AL* removal is due to the magnifying effect by nicotine on a sudden increase in cue reinforcing effects, whether primary or secondary in nature. Supporting this view, previous studies show that nicotine can increase the reinforcement and incentive salience of cues that have already reinforcing value, whether primary or secondary (Donny et al., 2003; Chaudhri et al., 2006; Palmatier et al., 2007, 2013; Rupprecht et al., 2015). It thus follows that any increase in salience of nicotine-paired cues would be magnified even further by nicotine, as supported by our study. No other study to date has specifically addressed this possibility. By comparison, decreasing the cue salience by introduction of the *AL* has the same decreasing effect on both *nicotine + cue* and *saline + cue* self-administration, suggesting in this instance a non-specific decrease in visual perception, which is not altered by nicotine.

### Varenicline Targets the Reinforcing Effects and Reinforcing-Enhancing Effects of Nicotine on Its Associated Cue

In accordance with the literature (Rollema et al., 2007b; O’Connor et al., 2010; Le Foll et al., 2012; Funk et al., 2016), we showed that Varenicline 1 mg/kg reduces *nicotine + cue* self-administration. We were interested in exploring whether such robust decrease in self-administration is due to Varenicline affecting nicotine reinforcement, nicotine-cue interactions, or a combination of both. Here we demonstrated that acute Varenicline also decreases behavior in rats self-administering nicotine alone, although to a lesser absolute extent. In the same conditions, acute Varenicline has no effect on the self-administration of the salient visual cue by itself.

A limitation in exploring Varenicline effects on the sole reinforcing effects of nicotine is that these are relatively weak, and even for those rats that acquired nicotine self-administration without the presence of a nicotine-paired cue, their baseline nicotine-seeking behavior is substantially lower than for *nicotine + cue* self-administration. This could compromise the detection of Varenicline effects, as decreases in responding are less evident when the baseline responding is already low. In trying to bypass this limitation, a recent article by Kazan and Charntikov (2019) studied the role of Varenicline in nicotine reinforcement through a behavioral economics approach. Briefly, they trained rats to self-administer *nicotine + cue* through daily escalated FR schedules of reinforcement, calculated the individual baseline demand for nicotine, and assessed the individual effect of Varenicline as a function of nicotine demand. They show that individual demand for nicotine predicted the individual reduction in self-administration after a Varenicline challenge. This could look contrary to our results (i.e. absence of correlation between basal self-infusions and Varenicline effect on basal self-administration in the *nicotine* group - experiment 1) because escalation of schedules of reinforcement is supposed to bring into evidence the role of nicotine reinforcement. However, the *nicotine + cue* protocol used by Kazan and Charntikov (2019) cannot disentangle the primary reinforcement of nicotine from the reinforcement-enhancing effect of nicotine on the associated visual stimulus. The same protocol with nicotine as the sole reinforcer would help clarify the case.

Our study also complements previous findings in clarifying the reinforcing-enhancing effects of Varenicline on a visual cue: namely, that these effects are only observed when individuals have been previously exposed to nAChR agonists. Contrary to our study, Clemens et al. (2017) and Barrett et al. (2018) showed that acute Varenicline increased the self-administration of a visual cue alone in the absence of nicotine. Furthermore, Levin et al. (2012) briefly reports in drug-naïve animals, the reinforcing-enhancing effects of Varenicline on visual cues. However, and differently to our case, in these studies, rats had been previously exposed to either nicotine or Varenicline. In Clemens et al. (2017), rats had been previously trained for *nicotine + cue* self-administration and Varenicline tested after seven self-administration of the cue alone, through a nicotine extinction-like procedure. In Barrett et al. (2018), Varenicline was tested following a history of repeated passive exposure to nicotine administered after the cue self-administration sessions. In Levin et al. (2012), the authors make a brief comment that the reinforcing-enhancing effects of Varenicline were evident in the first seven sessions of repeated Varenicline exposure, although it remains unknown if the reported effects were already substantial during the first session. It is noteworthy that in these three cases, the reinforcing-enhancing effects of Varenicline appear similar, regardless of whether the nicotinic agonist was present at the moment of cue self-administration (Levin et al., 2012; Clemens et al., 2017) or disconnected from it (Barrett et al., 2018). In our study, the lack of previous history with nAChR agonists in *saline + cue* rats could thus explain the lack of previously described reinforcing-enhancing effects of Varenicline (Levin et al., 2012; Clemens et al., 2017; Barrett et al., 2018). This temporal requirement could most probably involve upregulation of α4β2-containing nAChRs, caused by chronic exposure to both nicotine (Marks et al., 1983; Buisson and Bertrand, 2001; Staley et al., 2006) and Varenicline (Marks et al., 2015). Nicotine, however, is known for its acutely reinforcing-enhancing effect of stimuli, even in drug-naïve individuals (Rupprecht et al., 2015; Perkins et al., 2017). This supports that Varenicline does not necessarily reproduce a nicotine-like increase in cue reinforcing effects, but requires a cholinergic system already sensitized to nicotinic agonists, which makes rats more sensitive to the reinforcing-enhancing effect of nicotinic agonists to cues. In addition, within the same study by Levin et al. (2012), Varenicline 1 mg/kg both failed and succeeded to increase the reinforcing effects of a visual stimulus in two distinct experiments with similar design, obscuring any consistent interpretation of the effect of Varenicline at this dose. Possibly, the effect of varenicline in enhancing the reinforcement of visual stimuli could be better seen at lower varenicline doses, as reported by Levin et al. (2012), which we failed to observe in this study. Further studies using different varenicline doses are needed to explore this possibility.

### Varenicline Targets the Reinforcement-Enhancing Effect of Nicotine on Its Associated Cue During Self-administration

Using a novel visual interfering procedure, we evidenced that Varenicline appears to specifically reduce the reinforcement-enhancing effects of nicotine on surrounding cues during nicotine self-administration.

Varenicline effect on nicotine self-administration was bi-directional, depending on how individuals responded to the manipulation of the *AL*: the more *AL* removal increased self-administration, the stronger the effect of varenicline in opposing cue salience (Figure 4C), while the less AL insertion decreased self-administration, the stronger the effect of varenicline in decreasing cue salience (Figure 4F). This correlation was stronger for the *AL* removal condition. It is possible that the weaker correlation in the *AL* insertion condition is related to a lower number of rats tested. Nevertheless, these results add to the evidence shown by Kazan and Charntikov (2019), that Varenicline’s effects appear dependent on individual differences in nicotine reinforcement. To our knowledge, we are the first to report an effect of Varenicline that is dependent on the strength of nicotine-cue interactions: a stronger nicotine-cue interaction is associated with a stronger Varenicline effect. This observation supports the rationale for individual variations in the mechanisms of nicotine-seeking (Garcia-Rivas and Deroche-Gamonet, 2019), with some individuals being more sensitive than others to the influence of the reinforcement-enhancing effect of nicotine on environmental cues, and who could differently benefit from Varenicline treatment.

It has been previously shown that the reinforcement-enhancing effect of nicotine on cues is not only dependent on α4β2-containing nAChRs (Liu et al., 2007), but also on the dopaminergic system (Palmatier et al., 2014). Given the precise molecular pharmacology of Varenicline, a possible mechanism for Varenicline could be antagonism at the α4β2-containing nAChRs located in the ventral tegmental area (VTA), thus reducing the nicotine-induced tonic firing of dopaminergic neurons, leading to decreased tonic release of dopamine in the nucleus accumbens (NAcc; Crunelle et al., 2010). Such a mechanism could also be involved in the effect of Varenicline on the primary reinforcing effects of nicotine, which are also thought to be dependent on VTA to NAcc signaling (Di Chiara, 2000; Picciotto and Corrigall, 2002). However, acute Varenicline appears to target the former, as a function of individual response, but not the latter. An alternative mechanism could involve α7 nAChRs, or other structures in the circuitry controlling nicotine-cue interactions, such as the basolateral amygdala, an area rich in α4β2- and α7 nAChRs (Feduccia et al., 2012) and also involved in drug-cue interactions (Janak and Tye, 2015).

In our study, we have investigated the psychopharmacological targets of Varenicline during early *nicotine + cue* self-administration. Future studies should address whether prolonged exposure to nicotine changes the way Varenicline affects *nicotine* and *nicotine + cue* self-administration. The differential effects of Varenicline in *nicotine + cue* self-administration in short vs. prolonged exposure to nicotine might depend on the experimental approach: George et al. (2011) reports that Varenicline does not differently affect rats with long access to nicotine (23-h sessions) compared to short access (1-h session). The study by Clemens et al. (2017) on the other hand, shows that after an extended training (40 sessions) with a short access protocol, Varenicline seems to also target the reinforcing properties of nicotine alone, compared to early training (20 sessions). However, the specificity of this Varenicline effect is problematic, as the decrease is seen both in active and inactive responding. These results warrant further exploration.

Furthermore, as a treatment for tobacco cessation, daily doses of Varenicline are recommended in the week leading up to a cessation attempt, with continuous daily administration over the following 11 weeks after cessation (Ebbert et al., 2010). While our study only assessed the effect of an acute exposure to 1 mg/kg Varenicline, further studies need to assess if prolonged exposure to Varenicline affects the psychopharmacological dimensions of nicotine-seeking during nicotine self-administration in a different way than those after acute exposure. Studies with repeated Varenicline administration have been performed but focused on the reinforcing effects of a visual cue either in rats never exposed to nicotine (Levin et al., 2012) or previously administered with passive nicotine injections (Barrett et al., 2018).

Despite this, our results raise therapeutic implications. Increasing clinical and preclinical data suggests that smokers differ in the mechanisms that drive their nicotine-seeking (Garcia-Rivas and Deroche-Gamonet, 2019), with some smokers having stronger sensitivity to the primary reinforcing actions of nicotine (Hutchison et al., 2007; Esterlis et al., 2016), while others being more sensitive to the effects of nicotine on surrounding cues (Perkins, 2009; Perkins et al., 2017; Van Heel et al., 2017). Our results support individual variations in both nicotine reinforcing effects and nicotine-induced enhancement of cue reinforcing effects in the rat. Our data also suggest that individual variations in nicotine-induced enhancement of cue reinforcing effects, but not individual variations in nicotine reinforcing effects, would determine the amplitude of acute Varenicline-induced decrease in seeking during volitional administration of nicotine. Altogether, Varenicline might be more beneficial for smoking cessation in those who are especially sensitive to nicotine effects on surrounding cues, and not for those who are more sensitive to the primary reinforcing effects of nicotine. Further studies need to clarify more precisely the action of Varenicline, using a preclinical model that would allow for the fine exploration of individual differences in the mechanisms that drive nicotine-seeking (Garcia-Rivas et al., 2017).

## Data Availability

The datasets generated for this study are available on request to the corresponding author.

## Ethics Statement

All procedures involving animal experimentation and experimental protocols were approved by the Animal Care Committee of Bordeaux (CEEA50, N° 50120168-A) and were conducted in accordance with the guidelines of the European Union Directive 2010/63/EU regulating animal research.

## Author Contributions

VG-R, NC and VD-G designed the experiments. VG-R, J-FF, NC, MC-G, PR and JT performed the research. VG-R, J-FF, NC and VD-G analyzed the data. VG-R and VD-G wrote the article.

## Conflict of Interest Statement

The authors declare that the research was conducted in the absence of any commercial or financial relationships that could be construed as a potential conflict of interest.
