# Supplementary material for: Varenicline Targets the Reinforcing-Enhancing Effect of Nicotine on Its Associated Salient Cue During Nicotine Self-administration in the Rat
Source: Front Behav Neurosci. 2019 Jul 17;13:159. doi: 10.3389/fnbeh.2019.00159 (PMC6650579; doi:10.3389/fnbeh.2019.00159)
Supplement: Supplementary file 1 [file Data_Sheet_1.pdf]

Supplementary Figure 1

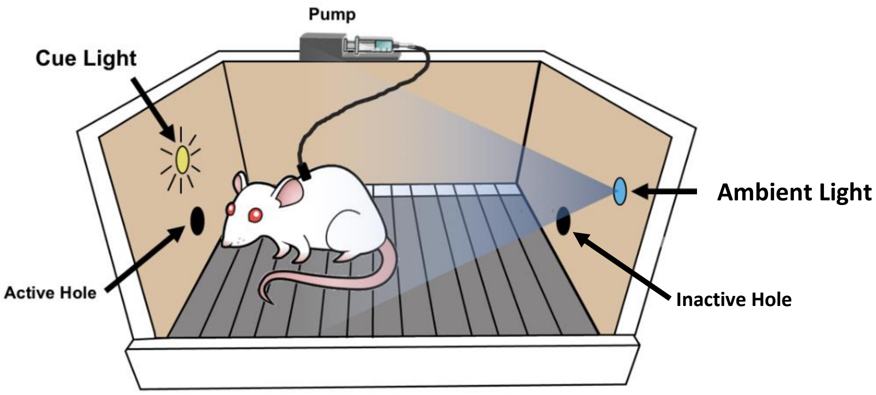

Scheme of the intravenous self-administration operant chamber. Each chamber (40 cm long x 30 cm width x 36 cm high) was located in an opaque sound-attenuating cubicle equipped with an exhaust fan to assure air renewal and to mask background noise. For self-administration sessions, each rat was placed in one chamber where its chronically implanted intravenous catheter was connected to a pump-driven syringe (pump). Two holes, located at opposite sides of the chamber at 5.5 cm from the grid floor, were used to record instrumental responding. In given experimental groups and experiments, a white light (white LED, Seoul Semiconductor, South Korea), 1.8 cm in diameter, located 11.5 cm above the active hole, was used as nicotine (or saline) delivery-associated discrete visual cue. A blue light (blue LED, Sloan Precision Optoelectronics, Switzerland), 1.8 cm in diameter, located on the opposite wall at 17 cm of the floor on the left side, was used as ambient light. It produced 15 Lux at a wavelength of 470nm.

Supplementary Figure 2

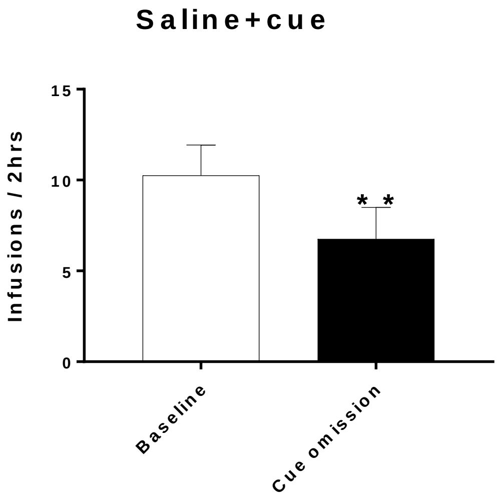

Cue omission decreases *saline+cue* self-administration. Mean infusions earned in basal conditions (*Baseline*) and during a cue omission test in rats self-administering *saline+cue*. For *Baseline*, infusions are averaged over the two last sessions prior to omission test. **p<0.01. Bars denote group mean and error bars denote SEM.

Supplementary Figure 3

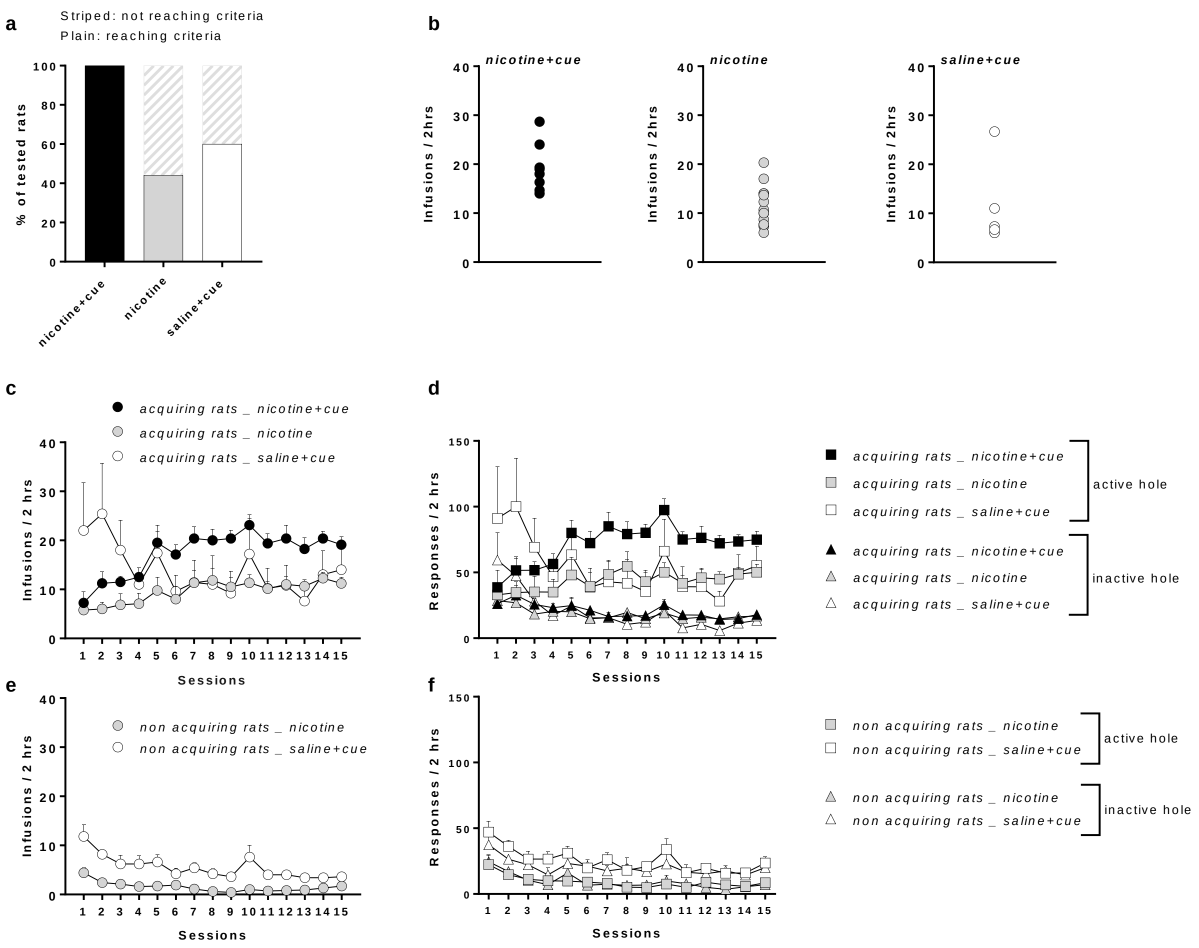

Acquisition of self-administration behavior by session 15 in rats self-administering *nicotine*, *nicotine+cue* or *saline+cue*. **a.** Percentage of tested rats acquiring self-administration according to the defined criteria. **b.** Distribution of individual scores of mean self-infusions per session in rats meeting acquisition criteria, in *nicotine+cue*, *nicotine*, and *saline+cue* groups, from left to right. **c-d.** Mean self-infusions per session and hole responses (squares=active hole, triangles=inactive hole) in rats meeting self-administration criteria. **e-f.** Mean self-infusions per session and hole responses (squares=active hole, triangles=inactive hole) in rats not meeting self-administration criteria, in the *nicotine* and the *saline+cue* groups.

c-f: Symbols denote group mean and error bars denote SEM.

Supplementary Figure 4

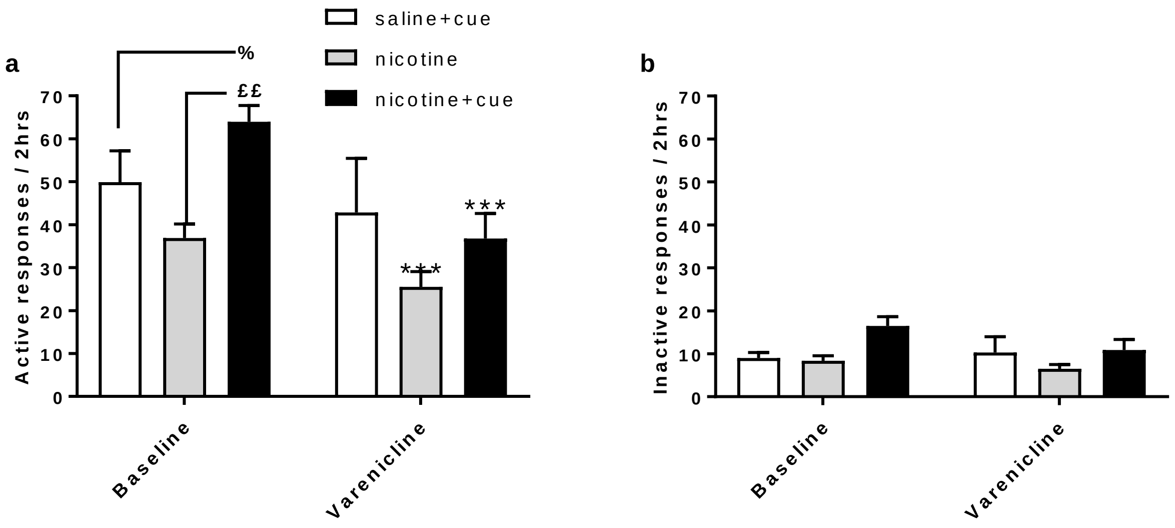

Varenicline targets the nicotine-cue synergistic interactions, rather than nicotine or cue primary reinforcing effects. a. Mean active nose-pokes in basal conditions (baseline) and after Varenicline administration (1mg/kg i.p., 30 min prior to session) in rats self-administering *saline+cue*, *nicotine+cue* or *nicotine*. For Baseline, responses are averaged over the two last sessions prior to Varenicline test. **b.** same as **a.** for inactive nose-pokes.

Bars denote group mean and error bars denote SEM. *** $p<0.001$ as compared to respective baseline. ££ $p<0.01$, % $p<0.05$.

Supplementary Figure 5

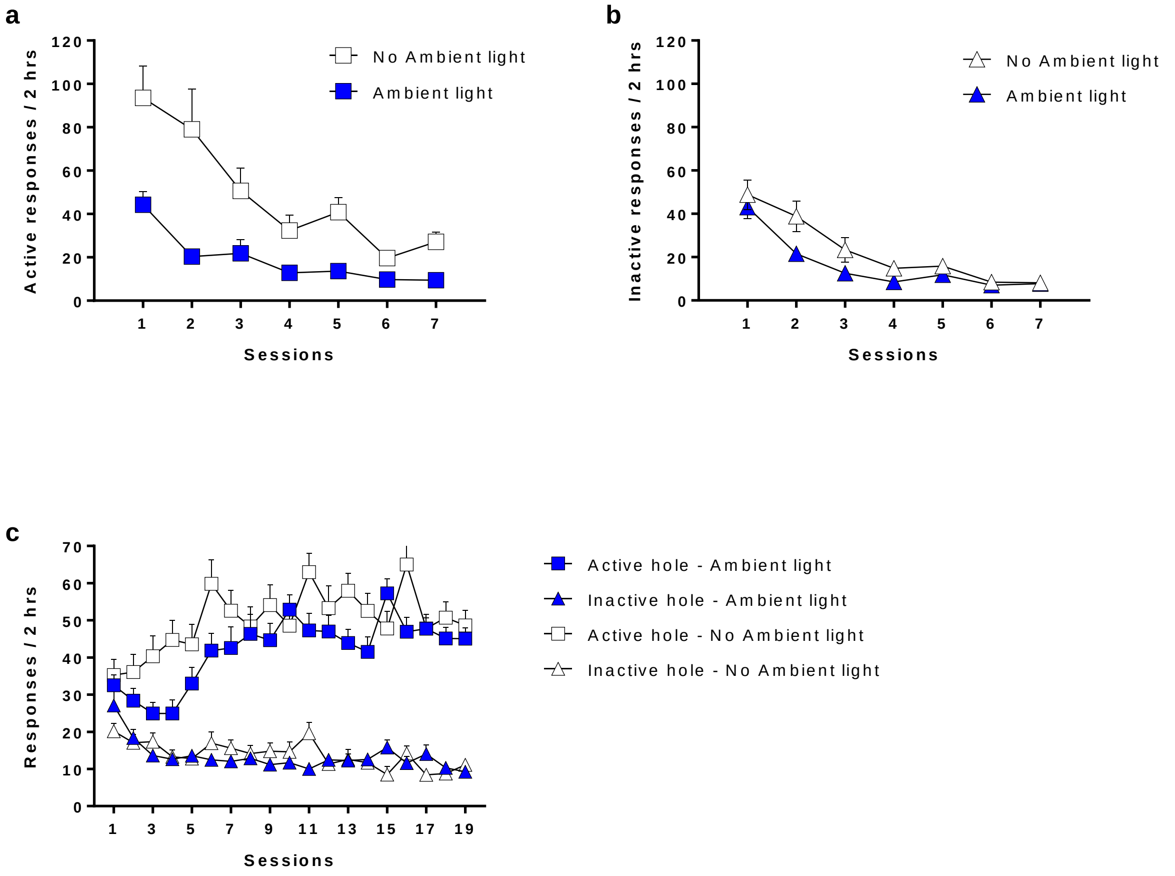

Impact of ambient light on *saline+cue* and *nicotine+cue* self-administration. a. Mean active responses per session in rats self-administering *saline+cue* in presence or absence of ambient light. **b.** Same as **a.** for inactive responses. c. Mean active and inactive responses per session in rats self-administering *nicotine+cue* in presence of absence of ambient light. Symbols denote group mean and error bars denote SEM.

Supplementary Figure 6

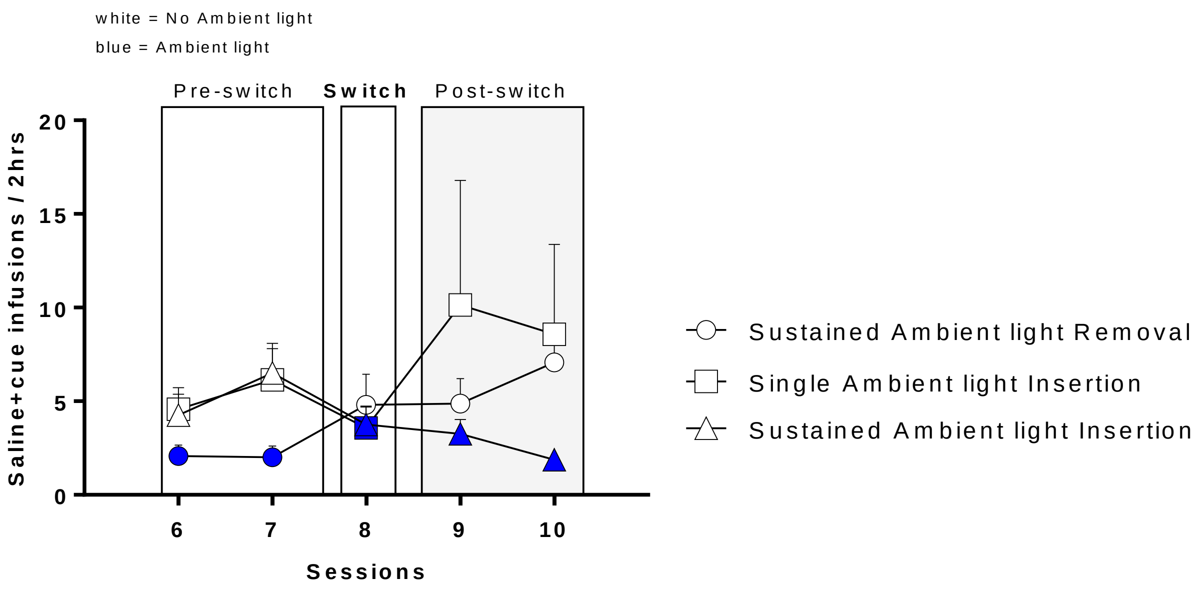

An interfering Ambient light alters of the primary reinforcing effects of a salient discrete cue light. Effect on *saline+cue* infusions per session of insertion or removal of an interfering **Ambient light**. Sessions 6 and 7 represent the last 2 of 7 self-administration sessions during which active nose-poking was reinforced at FR3 by the delivery of an intravenous infusion of saline associated with the lighting of a salient visual cue (*saline+cue*), in the absence (control - *No Ambient light* - white symbols) or presence (*Ambient light* - blue symbols) of an interfering Ambient light. At this point (session 8), according to **Figure 1B**, the Ambient light conditions were switched; turned off for the *Ambient light* group and on for the *No Ambient light* one (mean data shown on **Figure 3B**).

Then initial *No Ambient light* rats were split into two groups for the following two sessions (9 and 10): one group (*Sustained Ambient light Insertion*), maintained the newly acquired Ambient light condition, while the other (*Single Ambient light Insertion*) returned to their *No Ambient light* condition. Ambient light insertion effect was further amplified by *Sustained Ambient light insertion*, while a rebound was observed by Ambient light Removal in the *Single Ambient light Insertion* subgroup. To show the similarity in baseline and in response to *Ambient light Insertion* of the two subgroups of *No Ambient light* rats, results of the two subgroups are shown separately on sessions 6, 7 and 8 (white squares and triangles).

In the initial *Ambient light* rats, the removal of the Ambient light was maintained *(Sustained Ambient light Removal*), further increasing self-administration in comparison to sessions 6 and 7. Symbols denote group mean and error bars denote SEM.
